# Community effectiveness of pyriproxyfen as a dengue vector control method: A systematic review

**DOI:** 10.1371/journal.pntd.0005651

**Published:** 2017-07-17

**Authors:** Dorit Maoz, Tara Ward, Moody Samuel, Pie Müller, Silvia Runge-Ranzinger, Joao Toledo, Ross Boyce, Raman Velayudhan, Olaf Horstick

**Affiliations:** 1 Department of Public Health, Swiss Tropical and Public Health Institute, Basel, Switzerland; 2 University of Basel, Basel, Switzerland; 3 Public Health Specialist, Accra, Ghana; 4 Institute of Public Health, University of Heidelberg, Heidelberg, Germany; 5 Public Health Specialist, Ministry of Health, Brasilia, Brazil; 6 Division of Infectious Diseases, University of North Carolina at Chapel Hill, Chapel Hill, North Carolina, United States of America; 7 Department for the Control of Neglected Tropical Diseases, World Health Organization, Geneva, Switzerland; Faculty of Science, Mahidol University, THAILAND

## Abstract

**Background:**

Vector control is the only widely utilised method for primary prevention and control of dengue. The use of pyriproxyfen may be promising, and autodissemination approach may reach hard to reach breeding places. It offers a unique mode of action (juvenile hormone mimic) and as an additional tool for the management of insecticide resistance among *Aedes* vectors. However, evidence of efficacy and community effectiveness (CE) remains limited.

**Objective:**

The aim of this systematic review is to compile and analyse the existing literature for evidence on the CE of pyriproxyfen as a vector control method for reducing *Ae*. *aegypti* and *Ae*. *albopictus* populations and thereby human dengue transmission.

**Methods:**

Systematic search of PubMed, Embase, Lilacs, Cochrane library, WHOLIS, Web of Science, Google Scholar as well as reference lists of all identified studies. Removal of duplicates, screening of abstracts and assessment for eligibility of the remaining studies followed. Relevant data were extracted, and a quality assessment conducted. Results were classified into four main categories of how pyriproxyfen was applied: - 1) container treatment, 2) fumigation, 3) auto-dissemination or 4) combination treatments,–and analysed with a view to their public health implication.

**Results:**

Out of 745 studies 17 studies were identified that fulfilled all eligibility criteria. The results show that pyriproxyfen can be effective in reducing the numbers of *Aedes spp*. immatures with different methods of application when targeting their main breeding sites. However, the combination of pyriproxyfen with a second product increases efficacy and/or persistence of the intervention and may also slow down the development of insecticide resistance. Open questions concern concentration and frequency of application in the various treatments. Area-wide ultra-low volume treatment with pyriproxyfen currently lacks evidence and cannot be recommended. Community participation and acceptance has not consistently been successful and needs to be further assessed. While all studies measured entomological endpoints, only two studies measured the reduction in human dengue cases, with inconclusive results.

**Conclusions:**

Although pyriproxyfen is highly effective in controlling the immature stages of dengue transmitting mosquitoes, and–to a smaller degree–adult mosquitoes, there is weak evidence for a reduction of human dengue cases. More well designed larger studies with appropriate standardised outcome measures are needed before pyriproxyfen is incorporated in routine vector control programmes. Additionally, resistance to pyriproxyfen has been reported and needs investigation.

## Author summary

There is evidence that pyriproxyfen may effectively reduce the density of immature mosquito stages when applied to identified breeding sites. Various formulations are commercially available, and easy to use without a health threat to the user. However, questions remain regarding its use as a single agent in a community setting. Considering its mode of action, it would not be the product of choice for use in an acute outbreak setting. However, for a sustainable community approach, especially slow-release pyriproxyfen formulations seem promising, because they are the longest lasting choice. The analysis suggests, that combination with a second vector control chemical, preferably an adulticide tackling different stages of mosquito development, increases the efficacy of pyriproxyfen and prolongs the duration of a single application. This systematic literature review clearly shows that there is a need for further studies, preferably utilising cluster-randomised controlled (cRCT) designs, to investigate the community effectiveness of pyriproxyfen and to link entomological outcomes to human dengue transmission.

## Introduction

Over the past five decades, the global burden of dengue is estimated to have increased massively: Bhatt et al. [1] postulated that in 2010 there were 96 million apparent, and 294 million unapparent infections worldwide, with 22,000 registered dengue-related deaths reported in 2014 [2].

Transmission of dengue is through infective bites of female *Aedes aegypti (L*.*) (Diptera*: *Culicidae)* mosquitoes and, to a lesser extent, of *Ae*. *albopictus (Skuse)*. The immature stages of the *Aedes* mosquitoes are found in water filled containers [3].

In the absence of anti-viral medication and with the first commercially available vaccine not yet widely available for public health use [4], vector control remains the cornerstone for dengue prevention [5,6]. Due to their behaviour, adult mosquitoes transmitting dengue are difficult to attack and larviciding as well as larval source reduction are often the first choice of intervention. Larvicides have most often been implemented against *Ae*. *aegypti* as it breeds almost exclusively in domestic water containers. *Ae*. *albopictus* uses both, artificial and natural breeding sites [7] and is therefore more difficult to tackle.

Pyriproxyfen is an insect growth regulator (IGR) with a slow-acting larvicidal activity against a broad spectrum of public health insect pests [8] and it is being used extensively worldwide both in public and private settings. Acting on the endocrine system of insects by mimicking the juvenile hormone, pyriproxyfen hinders molting and subsequently inhibits reproduction. In addition, it causes morphological and functional aberrations in emerging adults, such as decreased fecundity and fertility. Due to its very low mammalian toxicity [9], pyriproxyfen is approved by the World Health Organization (WHO) for the treatment of potable water against mosquitoes [10].

Pyriproxyfen has been studied extensively in experimental, i.e. controlled laboratory or semi-field settings, with evidence of efficacy against immature *Aedes spp*. [11,12]. Yet, in field application studies, pyriproxyfen demonstrated mixed outcomes regarding its effectiveness as well as persistence. To date, no systematic review of the scientific literature has been undertaken to examine the evidence for the effectiveness of pyriproxyfen against dengue vectors. Therefore, the objective of this study is to review systematically the available literature for evidence on the community effectiveness (CE) of pyriproxyfen as a vector control method reducing *Ae*. *aegypti* and *Ae*. *albopictus* populations and dengue transmission.

## Methods

**Fig 1:**
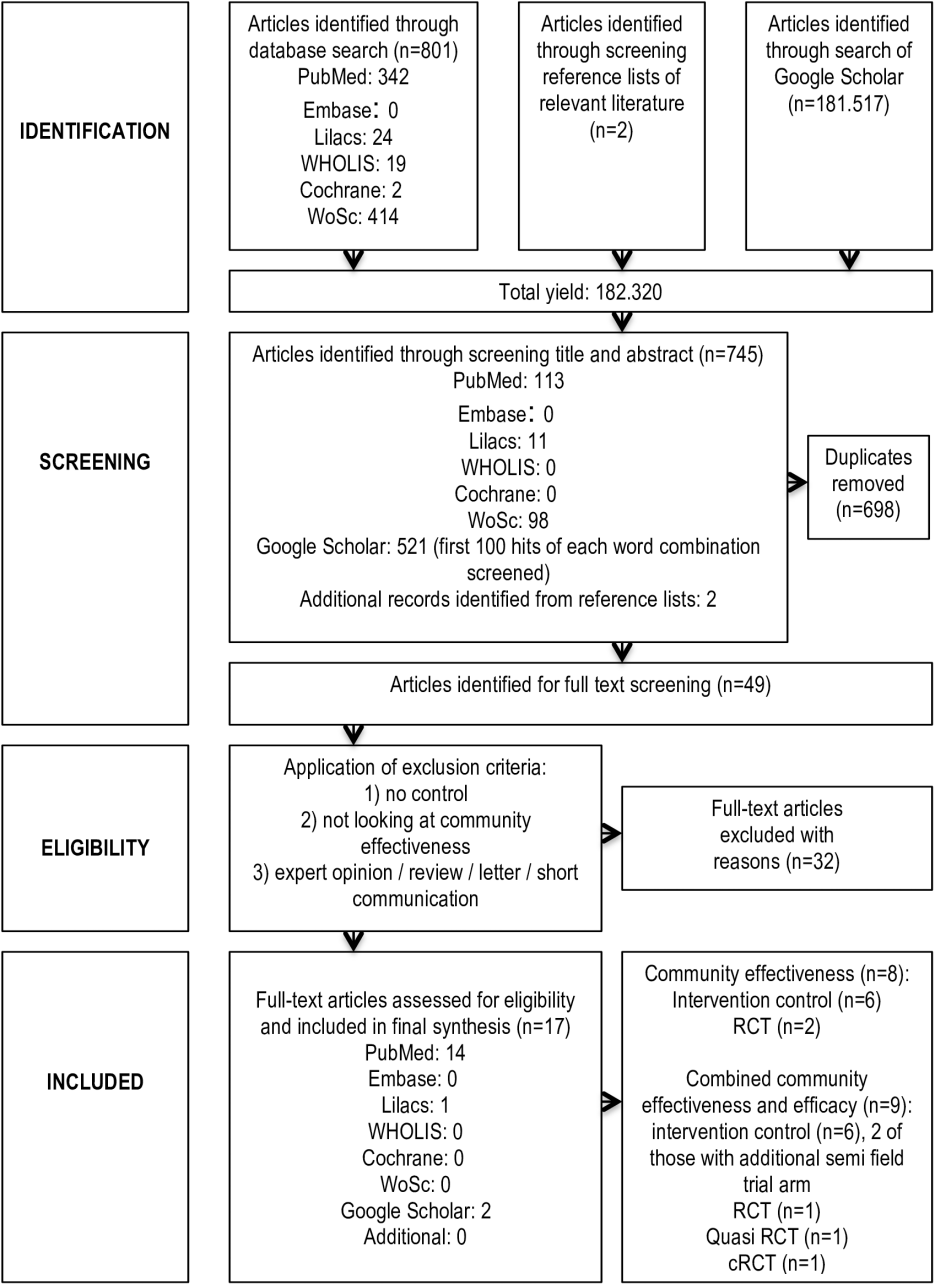
PRISMA flowchart of the selection process.

This review follows the reporting guidelines set forth in the PRISMA (Preferred Reporting Items for Systematic Reviews and Meta-Analyses) Statement for systematic reviews and meta-analyses [13] (Fig 1).

### Inclusion and exclusion criteria

The following inclusion criteria were used: 1) Studies providing original research dealing with the CE of pyriproxyfen—alone or in combination with other chemical vector control products. 2) As for study types, included were any cRCTs or randomised controlled trials (RCT); non-RCTs (nRCT) only if they were relevant to the research question and using a control, e.g. quasi-randomised controlled trials (quasi-RCTs), intervention control trials, controlled before and after studies. Unlike in RCTs, allocation in quasi-RCTs is performed in a way that is open to systematic bias, i.e. chances of being in one group or another are not equal. In cRCTs, pre-existing groups of participants are allocated to (or against) an intervention. Intervention control studies use methods designed to examine efficacy or effectiveness of an intervention in a group but do not use randomisation. 3) Any study that applied pyriproxyfen in the field—defined as any community or environment where dengue vectors naturally occur—was considered CE and included in the analyses.

Efficacy studies, defined as trials performed under laboratory conditions were excluded. Of the studies that undertook both methods, only the CE component was considered. Inclusion criteria included the above-mentioned study types, to give a broader picture of existing studies, since vector-control studies have varying designs and information may be useful.

### Search strategy

Two researchers independently carried out the literature search until 01 August 2016 with no starting time limit. The search was conducted in English, but articles were not excluded if the full text was not available in English. The search strategy was applied to the following seven databases to locate peer-reviewed studies: PubMed, Web of Science, EMBASE, LILACS, WHOLIS and Cochrane. In addition, grey literature using Google Scholar has been searched.

Search terms were divided into three broad categories, including 1) disease relevant terms, 2) vector relevant terms and 3) intervention relevant terms. For the disease category, the terms were: Dengue, Dengue haemorrhagic fever, Dengue shock syndrome, along with the abbreviations DF (dengue fever), DHF (dengue haemorrhagic fever), DS (dengue syndrome), and DSS (dengue shock syndrome); for the vector category: *Aedes*, *Aedes aegypti*, *Aedes albopictus;* and for the intervention category: pyriproxifen, and insect growth regulator.

Once screened for duplicates by author, title, journal and publication date, eligible studies were screened against the inclusion criteria. At first, titles and abstracts were screened by two independent reviewers, in a second step articles were reviewed in full and relevant information was extracted into the evidence table (Table 1). A third reviewer was available for the potential case of disagreement between the two independent reviewers. Studies were assessed for quality using the assessment of multiple system reviews (AMSTAR) [14]. For the purpose of this review, the overall quality of studies was not used to exclude studies, but as a tool for evaluating the impact of the reported outcomes.

**Table 1:** Evidence table.

1st author, year, country, vector	Study design	Objectives	Pyriproxyfen formulation / application	Intervention (a) / control (b) group	Duration F/U	Sample size / unit of allocation	Outcome measures	Results (only pyriproxyfen considered)	Conclusion
Caputo et al. 2012; Italy; Ae alb	int. control	Study the feasibility of an autodissemination approach to control Ae. albopictus in a highly urbanised neighbourhood	5% grinded to powder pyriproxyfen (Proxilar, I.N.D.I.A. Industrie Chimiche S.p.A.)	a) Verano Cemetery (98 ha) and enclosed garden of Sapienza University (1 ha) Sentinel sites with 200 ml tap water, 0.07 g of cat food and 25 3rd-instar Ae. albopictus larvae b) Control sites similar to SS but closed with nets to prevent mosquitoes from entering the beakers and transferring pyriproxyfen	12 days each in 2 consecutive years	2x 10 dissemination stations in 2 sites + either 10 sentinel sites or 10 control sites; the dissemination stations filled with tap water, covered with pyriproxyfen-dusted cloth	Pupal mortality in sentinel sites	50–70% (controls <2%); Mortality was always significantly higher in sentinel sites than in control sites and almost exclusively limited to the pupal stage	Pyriproxyfen was transferred by mosquitoes into sentinel sites and had a lethal effect. The study supports the potential feasibility of the autodissemination approach to control Ae. albopictus in urban areas.
Dantur Juri et al. 2013; Argentina; Ae aeg	int. control	To compare the efficacy of a ULV formulation and fumigant canister, containing both permethrin and pyriproxyfen, with that of standard permethrin applications in a field assay	Formulations (Chemotecnica S.A.Argentina): A: 10% permethrin as emulsifiable concentrate. 10 mL / ha. B: 3% pyriproxyfen plus 10% permethrin as emulsifiable concentrate; 100 mL / ha. C: fumigant canister with 1% permethrin + 0.2% pyriproxyfen. Fumigant components and inert c.s.p. 50 g. One tablet each 25–50 m^3^/10–20 m^2^ Zone A: Formulation A via cold fogger truck mount ULV Zone B: Formulation A via cold fogger truck mount ULV plus Formulation C employed at all dwellings Zone C: untreated control Zone D: Formulation B using cold fogger truck mount ULV E: Formulation A by portable aerosol generator ULV treatment	a) 64.591 people in five treatment areas; effects of A, B and C formulations were compared among 4 zones and among container types (250 ml and 20 l containers and adult cages) b) The fifth area was left untreated = control	10 weeks	Five areas (A—E) in the city, approx. nine blocks each, separated from one another by green areas / defined urban areas plus containers to measure the infestation rate in and around dwellings	BI; EI %; number of dead larvae; pupal survival	Treatment produced the most larval lethality; most larvae died after 2 weeks. The lowest BI (11) value was 1 week post-treatment in the area treated with formulation C but reached 118 by week 2; the next lowest BI (50), in the areas treated with formulation A, was stable for 1 week after application. After treatment, BI values of the area treated with formulation B took 3 weeks to recover, indicating better performance of this formulation. Comparing 250mL and 20L containers, container size seemed to influence insecticide dilution, which possibly affected EI%.	Permethrin + pyriproxyfen provides better (longer lasting) control of Ae. aegypti and could be a new alternative for vector control
Darriet et al. 2010; Martinique; Ae aeg	int. control	To evaluate the operational efficiency of a mixture of pyriproxyfen and spinosad against a population of Ae. aegypti from Martinique resistant to pyrethroid and organophosphate insecticides.	a) pyriproxyfen (0.02 mg/l), 3 with spinosad (0.1 mg/l) b) mixture of both (0.02 mg/l + 0.1 mg/l) c) controls	a) 1. step (simulated): evaluation of the efficacy of pyriproxyfen and spinosad when used alone, or in combination. 2. step: containers in natural conditions as above (only 2. step considered in this review) b) 3 untreated containers in same setting	7 months	15 containers	EI % / rate of adult emergence	1. step: the mixture of pyriproxyfen + spinosad remained active for minimum 8 months, compared with 3 months for spinosad alone, and 5 months for pyriproxyfen alone. 2. step: the rate of adult emergence was maintained at 20% for 3 wks (pyriproxyfen) / 3.5 months (spinosad). The mixture pyriproxyfen+spinosad remained effective for 4.5 months.	Combining the two larvicides with different modes of action increased the treatments residual activity.
Devine et al. 2009; Peru; Ae aeg	int. control	To demonstrate, in theory and practice that high coverage of aquatic habitats with a JHA is possible through treatment of only a small proportion of the adult resting area.	5 g pyriproxyfen/m^2^ (Sumilarv 0.5G; Sumitomo Chemical Corporation; a 0.5% granular formulation) pulverized to consistency of talcum powder	a) 10 pyriproxyfen dissemination stations, 40 uncontaminated sentinel oviposition sites with Ae. aegypti larvae b) controls and treatments were separated in time (same area pre and post treatment)	12 days F/U	3 trials at 2 sites with ten 1 l-plastic-pots in a public cemetery in Iquitos, Peru	EI 42–98% pupal/larval mortality up to 98%	The maximum mortality seen in individual trials was 98% and 59% at sites A and B, respectively. The effects were most apparent on non-feeding pupal stages. No loss of impact was seen on the sentinel site cohorts with increasing distance from the dissemination stations.	Adult Ae. aegypti are well suited for this technique because their resting sites have been well described and appropriate dissemination traps can be made accordingly. If adopted, the technique would need to be implemented within an integrated resistance management plan, probably involving the rotation or alternation of alternative control tools.
Doud et al. 2014; USA; Ae aeg and alb	int. control	To further evaluate the efficacy of pyriproxyfen delivered by truck-mounted ULV sprayer to control peridomestic container-breeding mosquitoes using laboratory-reared sentinel Ae. aegypti larvae and wild adult populations of Ae. aegypti and Ae. albopictus in St. Augustine, FL.	Nyguard H IGR EC (10% pyriproxyfen), provided by MGK Chemical Co. (Minneapolis, MN)	a) 1 plot of 40 ha, ca 200 houses (50-year-old family houses) b) 1 plot of 40 ha with no treatment	3 treatments from Aug 7—Sep 22, 2012, then further F/U for 2 wks	2 plots of 40 ha / 200 houses	EI %	Combined EI against Ae. aegypti larvae in sentinel cups in the field for spray activities 1, 2, and 3 were not significantly different (82%, 87%, 87%, respectively) Similar levels of EI achieved in both larvae when the measured field concentrations of pyriproxyfen were recreated in lab assays. Concentrations of deposited pyriproxyfen were sufficient to produce a mean EI ≥ 82% in sentinel colony-reared Ae. aegypti larvae.	This study for the first time demonstrates feasibility and efficacy of a truck mounted ULV application of pyriproxyfen at the scale of a city neighborhood. Pyriproxyfen was successfully deposited into sentinel cups up to 23 m from the ULV spray and even the smallest mean concentration among the distances from the spray line produced substantial mortality in sentinel larvae. The findings suggest that a truck-mounted application could be just as effective against Ae. albopictus larvae in the field
Harburguer et al. 2011; Argentina; Ae aeg	RCT	Evaluate the efficacy of an experimental fumigant formulation against Ae. aegypti in the field, and the residents’ acceptance of it together with its role in community participation for indoor control activities.	50-g fumigant tablet containing 10% permethrin, and 2% pyriproxyfen ULV: formulation containing 10% permethrin plus 2% pyriproxyfen as an emulsifiable concentrate (both by Chemotecnica S.A.) Polyethyleneglycol 1000 as antievaporant	a) Puerto Libertad—town of 6,000 residents on 2.62 km^2^—divided into four areas. One of four possible treatments assigned at random to each area. Two areas assigned for fumigant tablet (ft): one by the community itself (area A), the other by the local workers of the Nat. Vector Control Program (area B). In area C, professional ft application indoors was followed by truck mounted cold fog ULV treatment outdoors b) Area D had the same characteristics as A-C but remained untreated.	13 weeks	200 houses per each of four areas; field bioassays with 10 cages of adult Ae. aegypti randomly hung inside 10 dwellings (one / house) in areas B-D Ten 250 ml plastic jars with 15 Ae. aegypti larvae in tap water inside each dwelling of areas B-D cages and plastic jars placed 4–5 m from the ft, taken back to the lab after treatment, and followed up for EI	adult EI (90%) adult mortality (100%)	fumigant tablet (ft) alone: adult index fell to almost zero immediately after application and remained lower than control values for 8 wks. ft plus ULV: effect lasted an additional week; BI in this case decreased ca x30-40 compared with pre-treatment values and only regained values similar to the control area ca 8 wks after application > 80% of residents applied the ft and preferred participating in a vector control program by using a non- professional mosquito control tool, instead of attending meetings and workshops promoting cultural changes.	This study shows high penetration and mosquito adulticidal and larvicidal properties the ft. Same effect when applied by the community as when applied by professionals. Community is capable of participating in a mosquito control program with nonprofessional control tools, and does not consider a training workshop part of community participation. Application of ft plus ULV has a longer lasting effect (9 weeks) than ft only
Limpawitthayakul & Sornpeng 2011; Thailand; Ae aeg	int. control	To compare efficacy and sustainable killing effect of temephos with that of pyriproxyfen in the lab and in the field	Temephos 1% SG and pyriproxyfen 0,5% G	a) 4 villages	12 weeks	-	-	Temephos maintained its ability to kill 50% of larvae within 24hrs for 12 weeks in store water containers and 8 wks in used water containers. Pyriproxyfen 0.5% G acted longer: 13 and 9 wks, respectively. Most deaths at pupal stage or 3–15 days after water contact. In villages: similar effect shown	-
Lucia et al. 2009; Argentina; Ae aeg	int. control	To evaluate a new ULV formulation of permethrin + pyriproxyfen that is effective against adult and immature aquatic mosquitoes	Formulation A: Permethrin 15% as emulsifiable concentrate Formulation AL: (experimental) permethrin 15% plus pyriproxyfen 3% as emulsifiable concentrate (all by Chemotecnica S.A, Argentina) Polyethyleneglycol 1000 as antievaporant for ULV treatments.	a) Two areas were sprayed—one with pyriproxyfen 2g/ha + Permethrin 10g/ha, one with Permethrin 10g/ha only b) One area was left untreated	10 wks	2 areas of approx 36 ha each, plus 1 control. A total of about 100 houses with preexistent containers being used for intervention.	BI, HI, EI %	A: pretreatment levels after 3 weeks, lowest BI 69 one week after application AL: pretreatment levels after 5 wks, lowest BI 50 one wk after application, stayed low for 1 wk similar results for HI 1) Pyriproxyfen has no antagonistic effect on Permethrin 2) Permethrin acts immediately as an adulticidal 3) Pyriproxyfen acts as larvicidal	The effective control of A. aegypti adults and larvae suggests that the used formulation could be a new successful alternative for controlling dengue vector populations in open areas.
Marcombe et al. 2011; Martinique; Ae aeg	RCT	Characterise the resistance status of A. aegypti larvae from Martinique to conventional and alternative insecticides and assess their efficacy and residual activity in simulated and field conditions	Vectobac DT (1 tablet/50 liters, 5 mg/L) Sumilarv 0.5% GR (0.05 mg/L) Dimilin TB-2, 2% (1 tablet/200 liters, 0.2 mg/L) Spinosad DT (1 tablet/200 liters, 0.5 mg/L)	a) Three communes, each with 5 positive breeding sites selected for each single treatment with one of four insecticides: Bti, diflubenzuron, pyriproxyfen or spinosad. In total 20 intervention sites per community b) In each of three communes 5 positive breeding sites served as untreated controls	9 months	Three sites, ≥ 2 km apart: a) group of houses, 0.02 km^2^ b) classical housing estate, 0.12 km^2^ c) hamlet, 0.11 km^2^ Each site 5 randomly chosen breeding sites (various pre-existent containers)	EI %; RD = residual density of Ae. aegypti pre and post	A lower residual activity for all insecticides in field trials compared to semi-field trials was observed. Strong and significant difference of RD over time between treatment groups (P < 0.001). Mosquito RD strongly fluctuated over time and made estimating pyriproxyfens residual efficacy difficult. Pyriproxyfen lost its efficacy in the large field trial after 4 wks (RD >20%, EI <80%) EI rates for Bti and pyriproxyfen were > 95% two days after treatment, but rapidly decreased to 1.7% and 7.9% 28 days post-treatment.	Decreased susceptibility to pyriproxyfen may be due to cross-tolerance of larvae to IGR after extensive use of temephos in the past.
Ocampo et al. 2014; Colombia; Ae aeg	quasi RCT (int. control)	To describe a vector control strategy, its operationalization, the prioritization and control of breeding sites, and the subsequent entomological and epidemiological results, in an endemic town in southwestern Colombia	Each catch basin was treated monthly with 2 g of pyriproxyfen (approx 0.05 mg/mL)	a) Guadalajara de Buga, Colombia: 97.262 people, 32.224 houses b) neighboring town (Palmira) chosen as control for dengue case incidence	7 months	603 houses (indoors) in six communes 4800 street catch basins	BI; CI Stegomya indices from the entomological surveys in houses larval/pupal mortality dengue case incidence	phase 1: 1) most common type of potential breeding sites were ground level water storage tanks for laundry 2) outside, 58,3% of basins positive for Ae. aegypti 3) 100% of adult EI was observed with pyriproxyfen phase 2: 1) statistically significant reduction of Ae. aegypti in street catch basins 2) when the intervention stopped, dengue cases increased	1) highlights the importance of catch basins as Ae. aegypti breeding sites 2) high resistance to temephos shown > should not be used anymore 3) pyriproxyfen highly efficacious, no known cross resistance with temephos 4) reduction in +ve catch basins suggests an effect of pyriproxyfen on mosquito density and hence on larval infestation 5) targeting street catch basins in such areas (urban, endemic dengue) could help to decrease dengue transmission
Ponlawat et al. 2013; Thailand; Ae aeg	1 = int. control 2 = RCT	Evaluation of pyriproxyfen-treated devices on Ae. aegypti populations within an dengue-endemic village	Pyriproxyfen (Sumilarv 0.5G, Sumitomo Chemical Corporation, Osaka, Japan) at a 0.05 g AI/m^2^	a) Ban Chon village with 65 houses with 160 inhabitants b) Neon Mayom village (71 houses, 171 inhabitants) was untreated = control	30 weeks	Four random houses in the inner circle of the treatment village received one device/house BG-trap 1/house (4 houses in inner and 8 houses in outer area) Sentinel containers: 3 l plastic buckets with tap water placed randomly in ten selected houses in both inner and outer circles of each village CDC backpack aspirator collections in houses w/o BG-trap	only BG-sentinel trap count was significant: adult density parity %	step 1: Egg production was significantly reduced in females exposed to pyriproxyfen early in their development step 2: presence of the pyriproxyfen-treated device significantly reduced adult counts during the study period	This device can reduce fecundity and change the age structure of the Ae. aegypti population in the field. The device may suppress increases in Ae. aegypti populations Pyriproxyfen has a significant effect on egg production and deposition (and hence adult productivity) when females are exposed shortly after a blood meal
Seccacini et al. 2014; Argentina; Ae aeg	int. control + semi-field trial	To evaluate natural long-lasting materials containing pyriproxyfen to improve control strategies of Ae. aegypti	Chemicals: Paraffin, stearin and beeswax (Parafarm Saporiti, Argentina) Pyriproxyfen (China Kelinon Agrochemical Co., Ltd., China) Discs: 15 g of beeswax and paraffin/stearin 1:1 discs (9 cm in diameter) containing 10−1, 10−2, 10−3, 10−4 and 10−5% pyriproxyfen O-rings: 120 g of paraffin /stearin/sand mix (2:1:2) containing 1% pyriproxyfen	a) Three 200-l plastic water-storage tanks situated outdoors in a mostly shaded location b) One tank with two O-rings without pyriproxyfen was kept in a faraway place to avoid contamination	6 months	Three 200 l- plastic storage tanks with tap water and O-rings	EI %	Pyriproxyfen/S-mixture gave higher EI than beeswax The semi-field trial (with 200-l water-storage tanks) obtained 100% EI for 6 months (in lab for 1 yr)	effective adult EI with Pyriproxyfen decline in reproduction and reproductive failures delayed effects could further extend pyriproxyfens efficacy and residual effects advantages of long-lasting materials: long duration of efficacy, any breeding place can be made a larval ovitrap, easy to handle
Seng et al. 2008; Cambodia; Ae aeg	int. control	To evaluate an experimental “second generation” strand formulation of pyriproxyfen placed in naturally infested concrete water storage jars in a village setting between April and December 2005.	Pyriproxyfen 5% comprised of resin strands 3 mm in diameter and 40 mm in length (135 mg w/w containing 6 mg a.i. per strand	a) One side of the road households were chosen for intervention b) On the other side of the road, households were chosen as controls	34 weeks	366 households, 1,875 people 100 water storage jars in households	EI %	High retention of pyriproxyfen strands in jars Two weeks after treatment 99.8% EI in intervention area; remained >97% until week 22; until week 34 (= end of study) EI fluctuated btw 100–80.4% in control jars EI never above 3.5%	The experimental controlled-release pyriproxyfen formulation was effective against Ae. aegypti (>80% EI) in domestic concrete water storage jars for the duration of the 34 week study (= length of the main dengue transmission season)
Sihuincha et al. 2005; Peru; Ae aeg	int. control	To provide a comprehensive examination of the effects of a granular formulation of pyriproxyfen on field Ae. aegypti. To report on the effects of pyriproxyfen on 1) adult emergence from exposed 4th instars and pupae 2) egg eclosion, 3) use of adults as vehicles for transfer of lethal larvicide doses to previously untainted oviposition sites 4) effect of adult exposure on fertility and fecundity 5) repellency of pyriproxyfen to ovipositing adults 6) residual effects of pyriproxyfen applied to large storage tanks in the field	Pyriproxyfen formulation Sumilarv 0.5 G (Sumitomo Chemical Co., Osaka, Japan)—a granular formulation of 0.5% (AI) (wt:wt) (5000 ppm)	a) 16 tanks (200, 300, 600 l) in households b) control pots with tap water	5 months	16 tanks in households, positive for Ae. aegypti before intervention, in constant use as water sources for washing and bathing	larval/pupal mortality	88–96% mortality of larvae in water tanks for 5 months adult blood-fed females exposed to residues of 0.003 ppb can transfer enough pyriproxyfen to untreated sites to keep larvae from emerging no difference in number of eggs laid per female if treated with various pyriproxyfen concentrations or tap water presence of organic material (hay) in the water greatly increased the number of eggs laid	In the field: water collected from tanks treated with 50–83 ppb remained effective (88.0–96.0% mortality) for 5-months. Pyriproxyfen was effective at 0.01ppb (the lower end of the range reported for this compound) Exposure on the day of blood feeding led to drastically decreased eclosion of eggs As for pyriproxyfen transfer: 5 treated females per new site result in over 70% EI; length of exposure had no impact Ae. aegypti show no preference for ovipositing in nontreated water over pyriproxyfen treated water Pyriproxyfen is a highly effective larvicide with the potential to be an excellent intervention against dengue it is clear from our data on repellency and the horizontal transfer of pyriproxyfen that it might be possible to exploit adults as vehicles for dissemination
Snetselaar et al. 2014; Netherlands; Ae aeg	int. control	To describe the development of a new type of ovitrap, a multi-impact contamination device for Aedes mosquitoes, with the aim to create a user-friendly control device that does not rely on electricity or chemical insecticides. To demonstrate the potential adulticidal, autodissemination and larvicidal impacts of the agents deployed in the trap.	Pyriproxyfen (Chemos GmbH, Regenstauf, Germany) mixed with fungal spores and inert dust particles to create a dust mixture suitable for application on the gauze	a) 50 free-flying Aedes females b) Two plastic cups with tap water, 20 larvae and fish food, placed outside the experimental cage	18 days	50 free-flying Aedes females	larval mortality; EI %	Dissemination of pyriproxyfen led to >90% larval mortality in alternative breeding sites and 100% larval mortality in the trap itself, against a control mortality of around 5%	The trap also has an effect on breeding sites in its’ vicinity and could therefore be a useful tool in integrated vector management campaigns to provide protection around the house and in public places.
Suman et al. 2014; USA; Ae alb	int. control	To test the hypothesis that contaminated gravid females would transfer pyriproxyfen to new larval habitats.	NyGuard IGR concentrate -MGK Corp., Minneapolis, MN, USA) containing 10% pyriproxyfen, formulated as an emulsifiable concentrate. Pyriproxyfen was applied as per manufacturers recommendation (789.23 mL/ha). Pyriproxyfen showed LC50 = 0.012 ppb for Ae. albopictus.	a) Area-wide treatment intervention Keyport: 2027.3 people/km^2^, 105 ha Treatment and control sites encompassed >1000 parcels, with each parcel corresponding to a structure or house b) Area-wide treatment control site: Union Beach 181 ha (1384.7 people/km^2^)	6 weeks each in two consecutive years	14 BGS traps	BG sentinel traps: pupal mortality; EI %	Area-wide direct treatment efficacy was lower (30.3% in 2010 and 5.3% in 2011) than point-source treatments. Area-wide treatments were ineffective on field populations of Ae. albopictus monitored with BGS traps. Point-source treatment was conducted to determine efficacy in tires as well as a source for autodissemination. Differences in auto- dissemination in 2010 and 2011 can be attributed to higher rainfall in the 2nd year.	The study shows that ULV surface treatments of conventional formulation do not work for autodissemination High overall pupal mortality was achieved from directly treated tires sampled during 2010, although mortality was lower in 2011, possibly due to higher rainfall. The effectiveness of pyriproxyfen in auto-dissemination may be improved by developing specific formulations to treat vegetation and tires that can load high doses on mosquitoes.
Tsunoda et al. 2013; Vietnam; Ae aeg	RCT	To determine whether covering the lids of domestic water storage containers with OlysetW Net would help controlling Ae. aegypti To test the effectiveness of applying an IGR in flower vases and ant traps inside and around houses. To examine the abundance of immature Ae. aegypti before and after the intervention, and also compare the change in abundance between the trial and control areas	EcoBio-BlockS: neutralized cement (40.6% w/w), aggregate (pumice stone, 52.1% w/w), a mixture of aerobic bacteria and nutrient medium (4.1% w/w), and 0.5% granular formulation of pyriproxyfen (SumilarvW 0.5 G, Shinto Fine Co., Ltd., Osaka, Japan, 3.2% w/w). Content of pyriproxyfen in the block: 0.016% (w/w).	a) Tan Chanh commune, 313 trial households; 12.000 total residents b) 363 control households	5 months	flower vases, ant traps (pyriproxyfen) major types of water containers (ceramic jars, cylindrical concrete tanks, other concrete tanks, plastic drums, and plastic buckets), covered with Olyset Net (permethrin)	CI; HI pupae/container anti Dengue IgM & IgG	The Container Index in the trial area gradually decreased from Oct-Feb. The total CI in the trial area decreased in Oct and continued to be <20% until February. Number of pupae per container decreased for all three states of lid in the trial area in Oct.	Treatment by Olyset Net and pyriproxyfen had a strong negative effect on the prevalence of the immature Ae. aegypti, which persisted for at least 5 months after treatment. Seroprevalence rates were not significantly different between the trial and control areas. Number of positive containers and of pupae in the trial area were both less than in the control area after Olyset Net and pyriproxyfen treatment, suggesting that suppression of mosquitoes is due to the treatments tested.

A comparative analysis of the main study outcomes was conducted, using the quality of each individual study as a weighing tool. The use of randomisation, the calculation of sample sizes and the size of the unit of allocation all impacted the weight individual studies were assigned. Finally, analytical categories were developed based on the method of pyriproxyfen application. These categories were: 1) container treatment, 2) fumigation, 3) auto-dissemination, and 4) combination of pyriproxyfen with adulticides. As for 1), ‘container treatment’ is for the purpose of this review defined as any intervention performed by using any kind of *Aedes spp*. infested containers (Table 2).

**Table 2:** Table of categories. Note that these categories are not mutually exclusive. Some studies are included in more than one category.

Category	Description
Container Treatment	For the purpose of this review, defined as any intervention performed on any kind of *Aedes spp*. infested containers
Fumigation	Fumigant canisters are thermal active systems generating smoke; they have been shown to work against *Ae*. *aegypti*, (Masuh et al. 2003)
Auto-dissemination	In auto-dissemination, the insecticide is dispersed by the insects themselves. Adult *Aedes* topically contaminated with pyriproxyfen can transfer enough material to untreated water containers to exert a significant lethal effect on immature stages developing therein
Combination of pyriproxyfen with adulticides	Combining products with different ways of action can increase the intensity or the effect of an intervention. A precondition is that the combined products do not inhibit each other

## Results

745 articles were identified from the different databases for assessment (Fig 1). After removing duplicates (n = 698), 47 were left for closer analysis. An additional two studies were identified from the reference lists of the above. Applying full inclusion and exclusion criteria on these 49 studies, 17 met the pre-specified eligibility criteria. Eight of the studies were classified as CE studies, while nine reported both efficacy as well as CE components.

Common reasons for the exclusion of potentially relevant studies included the following: 26 studies reported only efficacy; one study was a meta-analysis without original data [15]; and for one study [16] the pyriproxyfen component of the intervention was not accepted by the community and therefore not analysed. Another CE study [17] had multiple study arms, but the arm in which pyriproxyfen was tested against *Aedes* was a simple intervention design without a control and was therefore excluded. Similarly, another study [18] described the use of pyriproxyfen against Aedes only as a simple intervention and did not use it in its RCT part. One proved to be a simple intervention [19] and one combined efficacy and CE study [20] was excluded as it did not use pyriproxyfen in its CE part.

### General study characteristics

The included studies were published between 2005 and 2014 (Table 1). One was in Thai the others in English. Seven of the studies were conducted in Central or South America (four in Argentina [21,22,23,24], one in Colombia [25], two in Peru [26,27]; four in Southeast Asia (one in Cambodia [28], two in Thailand [29,30], one in Vietnam [31]); two in the USA [32, 33], two in Martinique [34,35], and two in Europe (Italy [36], Netherlands [38]).

Information on potential confounding factors such as the socio-economic status of residents [23,25,28,31,33] or housing construction [22,28] was not systematically reported. Weather conditions, either historical or during the intervention period, were reported in six of the studies [26,28,31,32,33,35]. No study incorporated a specific economic analysis or provided cost estimates for consideration.

The most common study design was nRCT, used in 12 studies. Three were RCTs, one quasi-RCT and one cRCT. Regarding the vectors, *Ae*. *albopictus* alone was studied in two of the included studies [33,36], one study [32] tackled both, *Ae*. *aegypti* and *Ae*. *albopictus*, both laboratory-reared. The remaining 14 studies looked at *Ae*. *aegypti* only.

#### Study objectives and interventions

While all included studies had a CE element, some also examined the efficacy of pyriproxyfen [25,27], or resistance [35]. Others looked at different application forms, including auto dissemination [26,27,33,36,37], fumigation [22], ULV application [21,32], long-lasting materials [24,31], and controlled-release application [28,31]. Some tested the combination of pyriproxyfen with another active ingredient; seven of the eight CE studies used it either in combination with another product—permethrin [22,23,31], Beauveria bassiana [37]—or compared it to other products [21,29].

Among the studies with both CE and efficacy arms, three used pyriproxifen either in combination with spinosad [34] or temephos [29] or compared its effects with other vector control methods (temephos, diflubenzuron, *Bti*, spinosad; [35]).

#### Sample sizes and units of allocation

Various methods of pyriproxyfen application were used and information on sampling and reporting of sample size was often limited, making comparisons between the studies difficult. The largest study included communities with a total of 97,262 people [25], followed by 64,591 people [21]. Both studies were conducted in urban settings, facilitating a high coverage. Tsunoda [31] performed their intervention in a town of 12,000 inhabitants, while Marcombe [35] and Darriet [34] chose smaller settings, described as ‘three separate villages’, though the exact size population-wise is not specified. Two studies [27,30] were conducted in rural areas and reported a sample size of two villages (65/71 houses with 160/171 inhabitants) and 16 households, respectively. Likewise, [29] was undertaken in four villages in Thailand.

The included studies also differed regarding the scale of the intervention; and, again, the reporting of this information varied widely. Sizes and makings of containers were most commonly reported as units of allocation. Others treated defined surface areas with different formulations and concentrations of pyriproxyfen. Some had more than one unit of allocation depending on method and study design. The smallest intervention was a cage with 50 free-flying *Aedes* mosquitoes [37], followed by [24] with 3 x 200 liter tanks and [34] with 15 containers of various sizes. The largest intervention [25] comprised the catch basins of a whole town (32.224 houses).

The follow up-periods—defined as the total intervention period from the first intervention day to the last day at which the endpoints were measured and reported—varied greatly. They ranged from 12 days [36] to nine months [35] with a median follow-up time of five months.

#### Outcome measures

The reported outcome measures differed depending on the respective research question and intervention (Table 3). The most widely used outcome measure, reported by 11 of the 17 studies, was per cent adult emergence inhibition (EI) [21,22,23,24,26,28,32,33,34,35,37].

**Table 3:** Summary of categorised results.

**Summary of container treatment studies**						
Study No	Pyriproxyfen			Max EI %	Main indices	Follow up time
	Formulation	Dose	Application			
Ocampo 2014	- (Government provided)	Approx 0.05 mg/ml	-	-	Positive catch basins / dengue incidence	7 months with monthly application
Marcombe 2011	Sumilarv 0.5% GR	0.05 mg/l	Granules in water	> 95	RD < 20%	4 weeks
Seng 2008	Pyriproxyfen	135 mg w/w containing 6 mg a.i. per strand	Resin strands 3 mm x 40 mm	99.8	-	34 weeks
Doud 2014	Nyguard 10% emulsifiable concentrate	164 ml/ha and 329 ml/ha	ULV spray	> 82	-	2 weeks
Lucia 2009	Emulsifiable concentrate 3% (plus permethrin)	2 g/ha (plus 10 g permethrin)	ULV spray	96	-	35 days
Suman 2014	Nyguard 10% emulsifiable concentrate	0.86 l/min	Point source treatment and area wide treatment	-	60.8 in 2010 (38.3 in 2011) % larval mortality	6 weeks each in 2 consecutive years
Dantur Juri 2013	3% pyriproxyfen plus 10% permethrin as	100 mL per ha	Emulsifiable concentrate	100	BI 72	3 weeks
Darriet 2010	0.02 mg/l	-	-	Fluctuating	RD fluctuating	21 days
Sihuincha 2005	Granular formulation 0.5%	50–83 ppb (= 10 mg/l)	Gauze bag in tank	‘Almost complete’	88–96% larval mortality	5 months
Seccacini 2014	1% long lasting pyriproxyfen		Paraffin/stearin/sand O-rings in tanks	100	-	6 months
**Summary of fumigation treatment studies**						
Study No	Pyriproxifen				lowest BI post-treatment	Time to pre-treatment BI
	Formulation		application			
Dantur Juri 2013	10% permethrin and 2% pyriproxifen		fumigant tablet		96 (down from 120) 4 weeks after treatment	8 weeks (9 weeks when combined with ULV)
Harburguer 2011	1% permethrin + 0.2% pyriproxifen		fumigant tablet		11	2 weeks
**Summary of autodissemination studies**						
Study No	Pyriproxyfen			Max EI %	Pupal / larval mortality	Follow up time
	Formulation		Dose			
Ponlawat 2013	0.5 G				0.05 g a.i./ m^2^	Only adult counts by BGS trap provided		13 weeks
Devine 2009	0.5% granular formulation, pulverised		5 g / m^2^	98	98	12 days
Caputo 2012	5%, grinded to powder		1 g per cloth (12x8 cm)	-	70	12 days
Snetselaar 2014	10% emulsifiable concentrate		798.23 ml/ha	-	10.3% in 2010 (2.9% in 2011)	6 weeks
Suman 2014	Pyriproxyfen by Chemos GmbH		-	-	> 90%	18 days
**Summary of combination product studies**						
Study No	Pyriproxyfen			Max EI %	Main indices	Follow up time
	Formulation	Dose	Application			
Harburguer 2011	10% permethrin and 2% pyriproxifen	2 tablets per house and 380 cubic cm every 36s	Fumigant tablet	-	BI 96 after 4 weeks (down from 120)	8 weeks (9 weeks when combined with ULV)
Tsunoda 2013	EcoBio-block S with 0.016% pyriproxyfen (plus permethrin as Olyset Net)	1 g/l of the 0.016% crushed pieces	Crushed EcoBio-block in containers		CI < 20% (from > 90%) IgM seroprevalence unchanged	5 months
Lucia 2009	Emulsifiable concentrate 3% (plus permethrin)	2 g/ha (plus 10 g permethrin)	ULV spray	96	-	35 days
Limpawitthayakul 2011	Temephos 1% SG and Pyriproxifen 0,5% G	-	-	-	Sustainable killing effect	9/13 weeks
Dantur Juri 2013	Emulsifiable concentrate 3% (plus permethrin)	100 ml/ha	ULV spray	100 in 250 ml; 20 in 20 l	-	3 weeks (250 ml)
Darriet 2010	Pyriproxyfen and/or spinosad	Pyriproxyfen 0.02 mg/l Spinosad 0.1 mg/l	-	Fluctuating	RD	21 days (pyriproxyfen), 4,5 months (mix)
Snetselaar 2014	Mix of B. bassiana plus pyriproxyfen	-	Dust mix applied on gauze	-	> 90% larval mortality	18 days

Regarding entomological indices, three studies reported Breteau Index (BI) only [21,22,25] and one BI and Container Index (CI) [23]. Pupae per person (PPP), currently considered the most highly associated with the density of adult vectors (36), was only reported by one study [23]. Pupal mortality [25,26,27,29,33,36], larval mortality [25,26,27,29,37], Relative Density [35], adult indices [22], and Container Index [31] were also used.

Only two studies linked human transmission data. One study [31] used serological surveys (Immunoglobulin M), Ocampo [25] measured dengue case incidence, drawn from the national reporting system.

### Analysis of container treatment studies

Ten studies [21,23,24,25,27,28,32,33,34,35] assessed the feasibility and efficacy of container treatment. Two studies were RCTs [25,35], the others intervention control studies. Four studies were performed in an urban environment [21,23,25,27], with the largest study covering an entire town [25]; the other six [24,28,32,33,34,35] were conducted in villages and rural areas.

In six of the studies [21,23,24,28,32,35], pyriproxyfen had a significant (82–100% EI) and long lasting (up to 8 months) effect. However, the two RCTs reported less positive results with significant effects for 4 weeks only. However, it must be considered that one RCT [25] measured catch basin positivity, making comparison with the other studies difficult. It also had several additional constraints such as moderate *Aedes* indices, low pupae/person indices from the start, inability to reach the intended sample size, and the emergence of a dengue epidemic during the intervention. The other RCT [35] and one nRCT [34] reported treatment persistence of only 4 weeks, though initial larval densities were significantly reduced.

One study [21] found a variable larvicidal effect in 20 containers and attributed this to larvicide dilution, though none of the other container studies reported efficacy limitations with increasing container sizes. Notably, Sihuincha [27] treated different sizes of water tanks successfully for five months despite a high turnover of the treated water.

Regarding area-wide ULV application, two studies [23,32] recommend this method of application over a wide range of container sizes, while one [33] found it not suitable for either larval habitat treatment or auto-dissemination. The two studies that did report good results with ULV application [23,32] used pyriproxyfen combined with permethrin.

As for persistence in container treatment, slow-release formulations had the best results. Sihuincha [27] reported a mortality rate >80% over five months with application by gauze bags, Seng [28] reported similar effects over eight months with resin strands and Seccacini [24] reported 100% EI at six months using pyriproxifen-impregnated O-rings. A shorter persistence of four weeks was reported from a large field trial with granules in water in Martinique [35], while the longest duration of effective EI through ULV treatment was 35 days [23], and the shortest only two weeks [32].

### Analysis of fumigation studies

Two studies [21,22] examined the use of fumigant canisters. Both used a combination of pyriproxyfen and permethrin and reported a significant inhibitory effect on adult emergence of *Ae*. *aegypti* as well as on BI. However, the size of the effect and persistence (less than 9 weeks) was limited. Adding outdoor ULV application of permethrin increased the effectiveness of the intervention.

### Analysis of auto-dissemination studies

Six studies evaluated auto-dissemination. The only RCT examining auto-dissemination [30] found significant results only in their BGS trap counts. However, these are known to be the most sensitive when counts are low from the start [39]. The remaining studies [26,33,36,37], using intervention control designs, primarily demonstrated that this approach is efficacious and that it can be applied easily and at low costs.

Caputo [36] performed field experiments with wild *Ae*. *albopictus* and reported an inhomogeneous product transfer to different sentinel sites. However, it was rightly pointed out that if the approach is applied to reduce *Ae*. *albopictus* adult densities, the mosquitoes themselves will disseminate the larvicide to the most attractive (i.e. most productive) natural breeding sites.

Devine [26] used 0.5% pyriproxyfen concentration in 1 l plastic containers and demonstrated overall reductions of *Ae*. *aegypti* adult emergence of 49–84%, as opposed to 7–8% in controls. In another experiment [36] with the same pyriproxyfen concentration, the overall reduction of *Ae*. *albopictus* adult emergence was 20.8%, as opposed to 2.4% mortality in controls. Given that the LC50 reported for *Ae*. *albopictus* (0,11 ppb; [39]) is about 10 times higher than that reported for *Ae*. *aegypti*, this is a promising result.

### Analysis of combination product studies

Seven of the reviewed studies combined pyriproxyfen with interventions targeting adults as well. They did not stratify the effects by product but rather as combined results. Five studies evaluated pyriproxyfen in combination with permethrin [21,22,23,29,31], one in combination with the fungus *B*. *bassiana* [35], and one with Spinosad [34].

Two of these studies were RCTs [22,31]. Both found significant changes through their respective interventions. While both used a combination of pyriproxyfen with permethrin, their application differed (fumigation versus EcoBio-block S) and so did their measured outcome values (BI versus CI and seroprevalence studies). Direct comparison is difficult but it can be summarised that they had good results that lasted for 9 weeks (fumigation plus ULV) and 5 months (EcoBio-block S), respectively. Similar results were reported in the non-RCTs.

In summary, the combination of adulticidal and larvicidal products can increase effectiveness by simultaneously controlling adults and larvae, and by expanding persistence [22].

### Resistance and combination treatments

Only one study found and reported on resistance of pyriproxyfen. Marcombe [35] demonstrated *Ae*. *aegypti* being tolerant against pyriproxyfen presumably due to cross-resistance with temephos, as pyriproxyfen had never been used on the island of Martinique before.

### Community participation and acceptance

Three of the included studies [22,25,28] described community perceptions and uptake of the interventions. Seng [26; Cambodia] reported overwhelmingly positive perceptions after an initial period of concern. Harburguer [22; Argentina] found the community capable and ready to participate in a mosquito control programme by using non-professional control tools (fumigant tablet). However, the authors also highlight the community’s reluctance to take part in training workshops, even though most applied the tablet while only 16% attended the workshop. The third study ([25]; Colombia) describes the importance of engaging and empowering local field staff regarding design and operation of entomological surveillance activities.

## Discussion

This systematic review of the CE of the juvenile hormone mimic pyriproxyfen against *Aedes spp*. presents evidence suggesting that pyriproxyfen can effectively control the adult emergence of immature stages of dengue vector mosquitoes in a variety of real world habitats. If the most productive breeding sites are identifiable and accessible–e.g. catch basins, water storage containers—direct treatment by monthly application appears to be the most effective and feasible with controlled-release formulations having strong and long-lasting effects.

With regards to efficacy, inconsistent results were presented [11,27]. These are most probably the result of differences between strains, formulations, and experimental conditions. There is a clear evidence that pyriproxyfen effectively inhibits *Aedes* adult emergence at concentrations of <1 ppb.

As to the methods of application, the evidence is highly variable. For container treatment, the effectiveness of pyriproxyfen seems to depend on factors such as the material of which the individual containers are made and the local environmental conditions. For example, Vythilingam [40] reported much higher levels of sustained residual activity in plastic tubs than in earthen jars, while Schaefer [41] and Glare [42] demonstrated a lower stability of pyriproxyfen at higher temperatures. In addition, there seems to be a natural inter-individual variation between containers [35]. There are contradictory reports on whether different container sizes play a relevant role, probably to be addressed by the amount of pyriproxyfen used.

Overall, the available evidence shows that a targeted treatment with a slow-release (e.g. granular) or a long-lasting (e.g. resin strands) formulation can yield an adequate EI for up to 34 weeks. However, more research is needed to define the lowest effective concentrations for each formulation and the frequency by which the treated containers should be replenished. Studies should be performed with standard containers in field trials to avoid accurate estimates of mosquito density being obscured by random variation among individual containers. Also, further work is needed to determine the impact of environmental conditions (UV light, dilution, temperature, etc.) on effectiveness and persistence.

Regarding ULV application, only interventions with a product combination (e.g. permethrin) showed a significant effect, suggesting that ULV treatment with pyriproxyfen alone cannot be recommended. Some authors discussed the potential reasons for the failure of ULV and hypothesised that a) container openings were too small for pyriproxyfen to enter; b) treatment surfaces were too small to cover a significant number of breeding sites; c) extreme weather conditions (first year very dry, second year higher than usual precipitation) adversely impacted the larvicidal effect; d) cryptic habitats of *Ae*. *albopictus* not accessed or e) the slow mode of action of pyriproxifen was not considered in the follow up evaluation [32, 33].

Space spraying–here as fumigation—has shown its effect only indoors, and it should be considered as part of a multi-intervention approach.

According to Devine [26], auto-dissemination could be an interesting approach to reach elusive breeding sites. Sihunicha [27] showed that an exposure of 30 min to water containing 0.003 g a.i./m^2^ pyriproxyfen allowed for horizontal transfer of effective larvicidal pyriproxyfen doses to untreated environments. The same study reports that subsequent eclosion of eggs was decreased by 70–90%.

Different ovitraps have been designed and their effectiveness tested. Extraordinarily low doses of pyriproxifen (*Ae*. *aegypti*: LC_50_ = 0.011 ppb (11), 0.012 [27], 0.0039 ppb [43]; *Ae*. *albopictus*: LC_50_ = 0.11 ppb [45]) are needed for this approach [27,44]. The herein reviewed studies reported maximum distances of 150 m (28 2013) and 200 m [45] travelled by the mosquito from the treatment sites. These are in line with Marini et al. [46] who demonstrated that gravid female *Aedes spp*. travel 50–200 m from the release sites. Kaufmann et al. [47] reported longer distances of up to 3 km. Further studies are needed to prove and improve the auto-dissemination strategy under field conditions. Specifically, methods of application, concentrations and frequencies of treatment need to be clarified.

Controlled-release/long-lasting formulations had a good effect with much longer persistence than other treatments. In order to improve adherence in community based approaches, such interventions should be preferred. They may even be more efficient as re-treatment would have to be less often and, therefore, the overall operation is more cost-effective as less professional personnel is involved.

Those studies that combined the application of pyriproxyfen with different adulticidal products clearly showed that effectiveness can be increased by simultaneously controlling adults and larvae, and by expanding persistence [22].

From the presented studies, there is insufficient evidence to determine what impact the level of motivation in a community could have on vector control. This question warrants further investigation in larger prospective studies. Such trials are also needed to assess whether and how communities can be motivated and control efforts sustained.

The majority of the included studies were from South and Central America, yet, Bhatt [1] postulates that Africa’s dengue burden is nearly equivalent to that of the Americas (i.e. 16 (11–22) million infections annually, representing, 16% of the global total). Also, India is estimated to contribute 34% (i.e. 33 (24–44) million infections per year) of the global total dengue infections. Further evidence is required on the effectiveness of pyriproxyfen in Africa and India to understand the influence of local environmental and societal factors.

Concluding, although pyriproxyfen is highly effective in killing the larvae of dengue transmitting vectors, and–to a smaller degree–also adult mosquito stages, evidence for the reduction of human disease transmission is weak. Lack of evidence is primarily due to small sample sizes, inappropriate study designs and lack of relevant, standardised outcome measures. Before issuing specific recommendations for the routine use of pyriproxyfen as a larvicide in dengue control programmes these research gaps must be addressed. Additionally, cross-resistance to pyriproxyfen has previously been reported by Macoris-Andrighetti [48] and, should it be verified, would have operational consequences for future dengue vector control. It therefore requires further attention in future studies as well as public health programmes.

## Supporting information

S1 Checklist PRISMA checklist for “Community effectiveness of Pyriproxyfen as a Dengue Vector Control Method: A Systematic Review”. (TIF)
