# Supplementary figures and images for: Community effectiveness of pyriproxyfen as a dengue vector control method: A systematic review
Source: PLoS Negl Trop Dis. 2017 Jul 17;11(7):e0005651. doi: 10.1371/journal.pntd.0005651 (PMC5531696; doi:10.1371/journal.pntd.0005651)

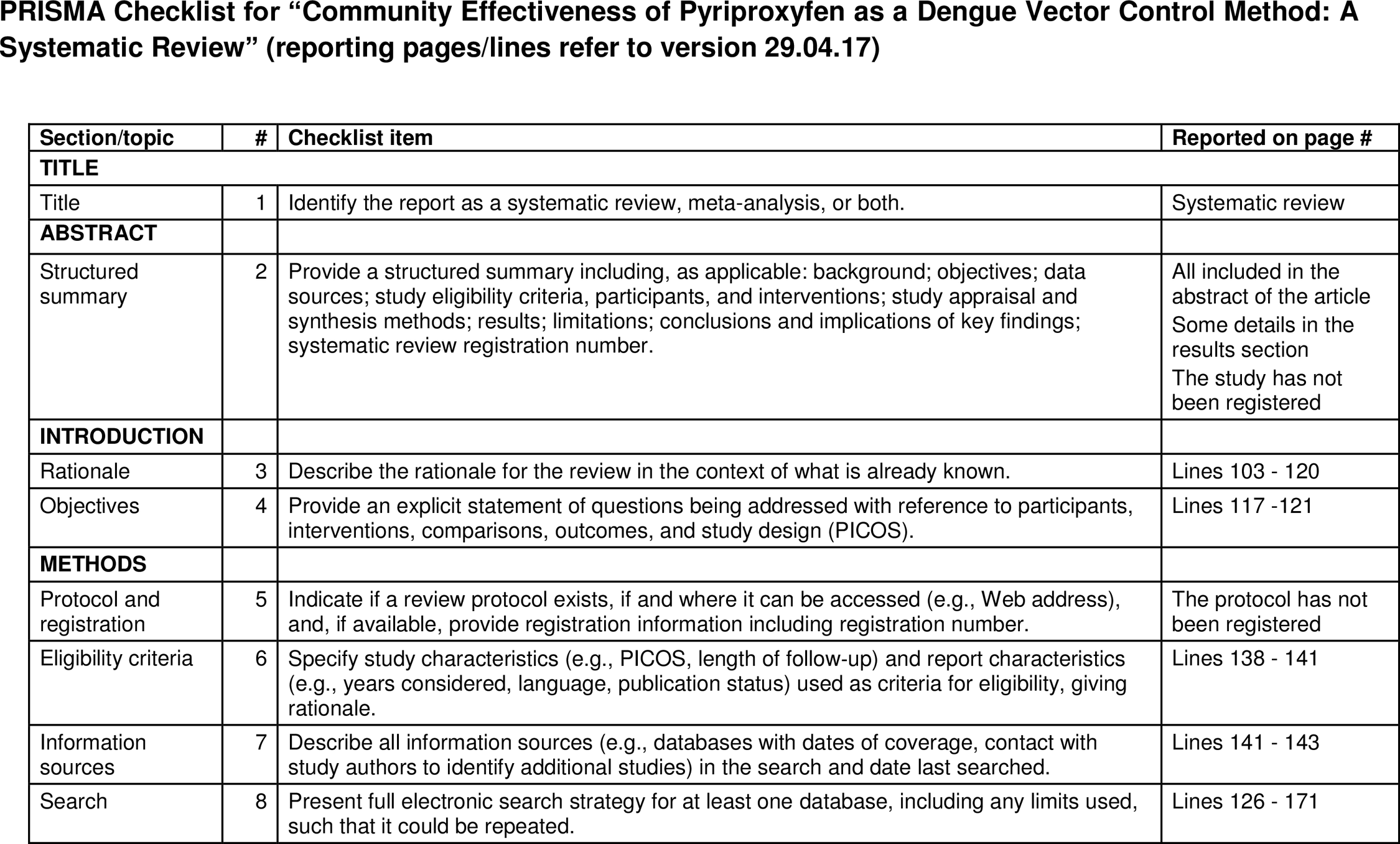

Supplement: S1 Checklist — (TIF) [file pntd.0005651.s001.tif]
